# Sex estimation from virtual models: exploring the potential of stereolithic 3D crania models for morphoscopic trait scoring

**DOI:** 10.1093/fsr/owad017

**Published:** 2023-06-23

**Authors:** Madeline Robles, Rachael M Carew, Carolyn Rando, Sherry Nakhaeizadeh, Ruth M Morgan

**Affiliations:** UCL Department of Security and Crime Science, University College London, 35 Tavistock Square, London, UK; UCL Centre for the Forensic Sciences, University College London, 35 Tavistock Square, London, UK; School of Applied Sciences, College of Health, Science and Society, University of the West of England, Coldharbour Lane, Bristol, UK; UCL Department of Security and Crime Science, University College London, 35 Tavistock Square, London, UK; UCL Centre for the Forensic Sciences, University College London, 35 Tavistock Square, London, UK; UCL Institute of Archaeology, University College London, 31-34 Gordon Square, London, UK; UCL Department of Security and Crime Science, University College London, 35 Tavistock Square, London, UK; UCL Centre for the Forensic Sciences, University College London, 35 Tavistock Square, London, UK; UCL Department of Security and Crime Science, University College London, 35 Tavistock Square, London, UK; UCL Centre for the Forensic Sciences, University College London, 35 Tavistock Square, London, UK

**Keywords:** forensic anthropology, virtual anthropology, 3D modelling, computed tomography, sex estimation

## Abstract

Modern computed tomography (CT) databases are becoming an accepted resource for the practice and development of identification methods in forensic anthropology. However, the utility of 3D models created using free and open-source visualization software such as 3D Slicer has not yet been thoroughly assessed for morphoscopic biological profiling methods where virtual methods of analysis are becoming more common. This paper presents a study that builds on the initial findings from Robles et al. (2020) to determine the feasibility of estimating sex on stereolithic (STL) 3D cranial models produced from CT scans from a modern, living UK population (*n* = 80) using equation 2 from the Walker’s (2008) morphoscopic method. Kendall’s coefficients of concordance (KCC) indicated substantial agreement using cranial features scores in an inter-observer test and a video-inter-observer test. Fleiss’ Kappa scores showed moderate agreement (0.50) overall between inter-observer sex estimations, and for observer sex estimations in comparison to recorded sexes (0.56). It was found that novice users could virtually employ morphoscopic sex estimation methods effectively on STL 3D cranial models from modern individuals. This study also highlights the potential that digital databases of modern living populations can offer forensic anthropology.

**Key points:**

- First example of Walker’s (2008) method applied to a living UK population.
- Open-source software is a valuable resource for crime reconstruction approaches.
- Male scoring bias was observed in method application.
- Forensic anthropologists would benefit from virtual anthropology training to use and interpret 3D models.
- Digital databases offer more ethical, diverse, modern populations for future research.

## Introduction

Historical skeletal collections and cemetery assemblages often act as a primary resource for forensic anthropologists in developing or testing biological profiling methods [1]. However, there are several drawbacks with relying solely on these collections. For example, these collections are not necessarily representative of contemporary (or indeed past) populations [1, 2], access to collections is extremely limited [3], and some raise ethical issues as a result of colonial antecedents and historical discriminatory practices [4]. The lack of appropriate, ethical and accessible collections consequently hinders the ability to test current methods used in forensic anthropology across forensically relevant modern global populations [3]. In recent years however, an alternative source for modern population data (derived from medical imaging databases) has been translated from its original medical purposes [5] for utilization in forensic anthropology.

There has been growth in the exploration of the use of 3D modelled bones from computed tomography (CT) data [6–10], and the use of medical imaging and virtual anthropology has been recognized as a suitable approach for developing and testing metric methods in forensic anthropology for direct applications to modern day populations [6, 7, 11–13]. However, there is little research that addresses the application and feasibility of forensic anthropological morphoscopic methods on 3D models of bones, which are arguably the most frequently used methods for sex and age estimations due to their ease of applicability [14, 15]. This study therefore further develops the work of Robles et al. [16] to determine the feasibility of estimating sex from virtual 3D cranial models (*n* = 80) using the macromorphoscopic (hereafter morphoscopic) trait scoring method presented by Walker [17] using eight observers with various degrees of experience in employing forensic anthropological methods and 3D modelling.

## Literature review

The use of modern imaging technologies to develop new approaches and methods within forensic anthropology applications is growing [7, 13]. Virtual 3D modelling of human anatomical structures has been established in forensic anthropology and is a tool that continues to be increasingly utilized [2, 6–10]. Although the accuracy of CT bone models has been confirmed in multiple studies [6, 18–20], virtual 3D modelling as a visualization approach is still in development [21]. Indeed, a large proportion of CT visualization platforms have been applied and tested within forensic anthropology, including commercial platforms such as Mimics, Amira, or Osirix [8], as well as free and open-source software, such as 3D Slicer or ITK-SNAP [22, 23]. In addition, the increased use of online platforms for training, teaching, and research is creating a new demand for the production and use of 3D models as primary teaching materials for anatomy, and forensic or biological anthropology applications [24, 25]. The increased use of online platforms and alternative teaching and research materials highlights the need for assessment of 3D models and their representation of anatomical structures.

However, the costs of licencing fees for commercial visualization programmes (such as Mimics and Amira), and additional maintenance fees [8] can prove prohibitive for funding bodies and public sector organizations that reduces the accessibility of these tools [21]. A study by Abdullah et al. [22] identified no significant measurable differences in the 3D models produced between commercial and noncommercial visualization platforms. However, to reliably implement free and open-source visualization platforms such as 3D Slicer, there is a need to fully assess their capabilities within forensic anthropology, where virtual methods of analysis are likely to become even more essential [16]. Bertoglio et al. [26] investigated cranial CT models for morphoscopic analysis and found that the models were good representations overall, but also identified limitations such as areas of missing bone, missing anatomic details, and misinterpretation of bone anomalies as pathological lesions. However, in their study the models from CT scans of dry bones were made, but only the volume renderings were then examined rather than a surface reconstruction made using segmentation [26]. Volume renders and surface reconstructions (including stereolithic (STL) models) are entirely different formats of 3D “models” that should always be explicitly identified to avoid misrepresentation. In terms of the issues identified related to missing bone or misinterpretation of anatomic details, Bertoglio et al. [26] suggested this could be resolved as software advances, however, such imaging anomalies will always be possible. Moreover, there is clearly a need for transparency in what specific models can achieve, and a place for training in medical imaging and 3D model reconstruction [27, 28].

The variability between populations has pushed studies to test the Walker method [17] across different populations [14, 29–31]. However, there are no published studies testing the Walker method [17] on a living UK population. Additionally, a number of studies have demonstrated the utility of CT data and/or 3D modelling and its pertinence in assessing morphoscopic differences to assist with sex estimation, such as for use with the maxillary sinus [32], the foramen magnum [33], or the pelvis [6, 34]. Considering cranial models specifically, 2D views and 3D volume reconstructions of the skull have been evaluated using general skull morphology [35] and using craniometrics [36]. Additionally, morphoscopic data have been obtained from volume renders [37]. However, the ability of anthropologists to utilize STL 3D models for traditional morphoscopic approaches (such as the Walker method [17]) and from UK modern population data is unknown. A step-by-step method for creating 3D models intended for those with minimal previous experience [16] has demonstrated the potential accuracy of models created from CT data by a range of users with a reproducibility within 1–2 mm. As a next step, these models need to be further tested to establish whether they can be reliably used in forensic anthropology applications. Therefore, the study presented here sought to determine whether it was possible to apply traditional morphoscopic forensic anthropology sex estimation methods on the STL 3D cranial models produced by Robles et al. [16].

## Material and methods

### Participants

**Figure 1:**
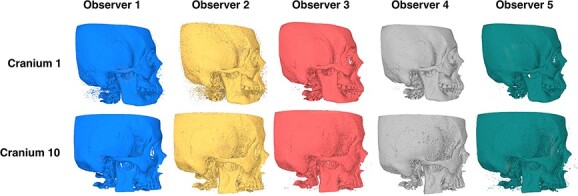
Matrix of two cranial stereolithic models (right lateral view) from all five observers in 3D Slicer.

In Robles et al. [16] STL cranial models were produced from 20 clinical sinus CT scans (10 male and 10 female) by five observers. The crania were from living individuals of mean age 54.5 years (male 26–91 years, female 29–64 years). The cranial models were reconstructed using 3D Slicer version 4.9.0 (Brigham Women’s Hospital, Boston, MA, USA) [38] following the method and scanning parameters outlined in Robles et al. [16]. Observers 1 and 2 had ⁓3 years of experience in 3D modelling and observers 3 and 4 had little to no prior experience. Observers 1–4 were all trained in forensic anthropology to master’s degree level or higher. However, Observer 5 was not familiar with applying forensic anthropology methods and was thus excluded from this study. Figure 1 illustrates two of the crania (cranium 1 and 10) modelled by each of the original observers. All participants provided written informed consent prior to data collection.

### Scores and equation

In this study, Observers 1–4 were asked to perform cranial trait scoring on the 3D models that they had created. Immediately after modelling, each observer re-loaded their STL cranial models into 3D Slicer using the “Data” module and scored the cranial morphoscopic traits using sex estimation methods based on Buikstra and Ubelaker [39] taken from Walker [17]. The CT scans were obtained for viewing sinuses and as such did not include the complete crania, and only three cranial traits were consistently observable and scored—the mastoid process, supra-orbital margin, and glabella. The Walker method [17] allows for fragmentary or incomplete skeletal elements to be used for sex estimations, as complete skeletal remains cannot be expected in forensic anthropological case work [17, 40].

Standard cranial trait scores of an ordinal scale of 1–5, as outlined by Walker [17] were used, with 1 typically representing more gracile (“female”) features, and 5 more robust (“male”) features. This method by Walker [17] was tested using American and British samples from the Hamann-Todd, Terry, and Saint Bride’s Church collections and is regularly used across various populations [14]. Sex estimations for each cranium were calculated using the cranial trait scores recorded by Observers 1–4 using logistic discriminant analysis equation number 2 from Walker [17] ($Y$=glabella$\times$(−1.568) + mastoid$\times$(−1.459) + 7.434), which eliminates some of the subjectivity around the scoring. The cut-off value to discriminate between a male and female sex estimation is a score of zero using the equation [14]. Equation 2 uses the glabella and mastoid and was the only equation suitable for use with the traits available for this study. The sex estimations derived from the cranial trait scores were compared against the known recorded sex of each cranium, with a percentage score for the number of correct classifications recorded for each observer.

### Video observer test

Four additional observers (video Observers V1–V4) were recruited to further assess the robustness of the models through a “video observer test”. The models were recorded using the screen recording function in QuickTime player ™ (.mov), where each cranium completed a 360° rotation about the lateral axis to provide full view of the cranial trait features in 3D Slicer. The full screen recording video was then shared with the video observers using a private link for the online platform YouTube. The incorporation of this “video observer test” created easy and remote access to the virtual models for four video observers to achieve a total of eight observers for this study.

The four video observers were forensic anthropology master’s degree students (two currently studying and two graduates) who each had no previous experience of using virtual 3D models. These video observers scored each of the 20 cranial models produced by Observer 1, using the same scoring and sex estimation method as outlined above. The models produced by Observer 1 were considered the “gold standard” for comparisons and all of the models produced were confirmed as metrically accurate to each other and verified for use [16].

### Statistical analysis

The level of inter-observer agreement was evaluated. Data were analysed using Minitab® version 17.1 for Windows and prepared using Microsoft Excel version 16.23 for Mac (Microsoft, Redmond, WA, USA). Fleiss’ Kappa [41] and Kendall’s Coefficient of Concordance (KCC) were employed, with the strength criteria from Landis and Koch [42] as a scale to assign agreement (as similarly used by Lewis and Garvin [14]): <0 = “poor”, 0–0.20 = “slight”, 0.21–0.40 = “fair”, 0.41–0.60 = “moderate”, 0.61–0.80 = “substantial”, and 0.81–1.00 = “almost perfect agreement”, meaning observer agreement is significantly greater than would be expected by chance. Kappa is appropriate for this dataset as it measures the degree of agreement for ordinal data (i.e. the cranial feature scores). Kappa is suitable for cases where multiple observers have assessed the same samples, and Fleiss’ Kappa (rather than Cohen’s Kappa) is used for more than two observers [43]. Additionally, Kendall’s coefficients take ordering into consideration that results in not all misclassifications being treated equally [43]. For example, Kendall’s coefficients consider that a score of 1 and 4 would have a higher degree of disagreement, than a score of 1 and 2. This ordering is appropriate for the cranial score data which are scored on a scale of 1–5.

## Results

### Cranial feature scores

The cranial feature scores from Observers 1–4 are presented in Table 1. In one case (Cranial 4) Observer 3 only scored the glabella. The results of KCC for each cranial feature were 0.68 for mastoid, 0.78 for supra-orbital margin, and 0.81 for the glabella, which indicated “substantial” to “almost perfect” agreement between the observers across the features using the Landis and Koch [42] classifications.

**Table 1:** Individual cranial feature scores for Observers 1–4.

Crania code	Mastoid process				Supra-orbital margin				Glabella			
	1	2	3	4	1	2	3	4	1	2	3	4
1	1	3	4	2	1	4	3	2	1	3	3	2
2	1	5	4	4	3	5	5	4	1	5	4	3
3	1	5	5	2	2	5	4	3	2	5	3	3
4	1	5	–	5	1	5	–	2	1	1	1	2
5	3	5	5	5	3	5	5	5	5	5	5	4
6	2	5	5	5	2	4	3	4	3	5	4	4
7	1	2	3	1	2	3	3	3	2	2	4	3
8	1	1	2	3	1	1	4	3	1	1	1	2
9	2	5	3	3	4	5	4	5	5	5	5	4
10	1	3	2	3	2	1	2	2	3	2	1	2
11	3	5	4	5	4	5	4	4	4	4	4	4
12	4	5	5	5	5	5	5	5	5	5	5	5
13	3	5	4	4	4	5	4	5	5	5	5	5
14	2	3	3	3	3	4	3	3	4	5	4	3
15	1	1	1	1	2	3	1	2	3	1	1	3
16	2	4	4	4	4	5	5	4	5	5	5	5
17	1	3	2	2	2	2	1	2	3	1	1	2
18	2	3	3	4	3	1	2	3	3	2	1	3
19	2	2	3	3	1	1	2	1	3	1	2	2
20	2	4	4	5	3	5	4	4	5	5	4	4

–: missing data.

**Figure 2:**
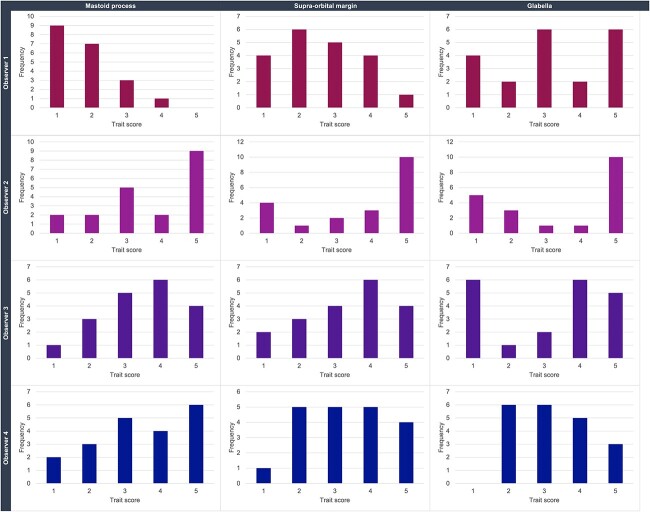
Bar charts illustrating the frequency of the cranial trait scores (1–5) per trait and per observer.

Frequency plots of the cranial traits scores (Figure 2) illustrate the distribution of the score data. Observer 1 assigned the mastoid process with low scores more frequently than the higher scores, and Observer 2 assigned scores of 5 more often than other scores across traits. Generally, the scores have varied distribution.

### Sex estimations

The cranial trait scores (Table 1) were used to obtain sex estimations using the Walker method [17] with equation 2 (Table 2).

**Table 2:** Sex estimation results for Observers 1–4 calculated from Walker (2008) equation 2 [17] (prob m/f = probability male/female) and recorded sex for each cranium.

Crania code	Observer 1			Observer 2			Observer 3			Observer 4			Recorded
	Sex	Prob m (%)	Prob f (%)	Sex	Prob m (%)	Prob f (%)	Sex	Prob m (%)	Prob f (%)	Sex	Prob m (%)	Prob f (%)	
1	Female	1	99	Male^a^	84	16	Male^a^	96	4	Female	20	80	Female
2	Female	1	99	Male^a^	100	0	Male^a^	99	1	Male^a^	96	4	Female
3	Female	6	94	Male^a^	100	0	Male^a^	99	1	Male^a^	55	45	Female
4	Female	1	99	Male^a^	81	19	–^a^	–	–	Male^a^	95	5	Female
5	Male	99	1	Male	100	0	Male	100	0	Male	100	0	Male
6	Male	55	45	Male	100	0	Male	100	0	Male	100	0	Male
7	Female	6	94	Female	20	80	Male^a^	96	4	Female	22	78	Female
8	Female	1	99	Female	1	99	Female	5	95	Male^a^	52	48	Female
9	Male	97	3	Male	100	0	Male	99	1	Male	96	4	Male
10	Female	22	78	Male^a^	52	48	Female	5	95	Male^a^	96	4	Female
11	Male	96	4	Male	100	0	Male	99	1	Male	100	0	Male
12	Male	100	0	Male	100	0	Male	100	0	Male	100	0	Male
13	Male	99	1	Male	100	0	Male	100	0	Male	100	0	Male
14	Male	85	15	Male	99	1	Male	96	4	Male	84	16	Male
15	Female	22	78	Female	1	99	Female	1	99	Female	22	78	Female
16	Male	97	3	Male	100	0	Male	100	0	Male	100	0	Male
17	Female	22	78	Female	18	82	Female	5	95	Female	20	80	Female
18	Male	55	45	Male	52	48	Female^a^	18	82	Male	96	4	Male
19	Male^a^	55	45	Female	5	95	Male^a^	52	48	Male^a^	52	48	Female
20	Male	97	3	Male	100	0	Male	99	1	Male	100	0	Male
% correct	95			75			65			70			

a Incorrect sex assessment.

Accurate sex estimations were obtained in 65%–95% of cases overall. Male crania were 90%–100% correctly estimated (average 95%), and female crania 40%–100% correct (average 58%).

### Video observer scores

Four video observers scored 20 crania each in the video test. Individual cranial scores from the video observers are presented in Table 3. KCC for each cranial feature were 0.79 for mastoid, 0.76 for supra-orbital margin, and 0.84 for the glabella, which indicated “substantial” to “almost perfect” agreement between the video observers.

**Table 3:** Individual cranial feature scores for video Observers V1–V4.

Crania code	Mastoid process				Supra-orbital margin				Glabella			
	V1	V2	V3	V4	V1	V2	V3	V4	V1	V2	V3	V4
1	2	4	2	2	2	4	2	3	3	3	3	2
2	4	5	3	2	4	4	2	2	4	4	2	2
3	3	5	2	3	4	3	3	4	5	3	1	2
4	5	5	2	2	3	2	1	3	2	1	1	1
5	5	5	5	4	5	5	5	5	5	5	5	5
6	5	5	4	4	4	4	2	3	5	4	3	3
7	1	2	1	1	3	2	1	4	3	3	2	3
8	3	2	1	1	2	2	3	2	1	1	1	1
9	3	4	4	2	5	5	5	5	5	5	5	5
10	4	4	3	3	3	2	1	3	3	3	2	2
11	5	5	5	4	4	4	3	2	4	4	2	2
12	5	5	5	4	5	5	3	4	5	5	5	5
13	4	5	4	4	4	5	5	5	5	5	5	5
14	3	4	2	3	4	4	3	3	4	4	2	1
15	1	3	1	2	3	2	1	4	3	3	2	2
16	4	5	4	3	5	5	4	5	5	4	5	4
17	2	3	3	2	3	2	2	3	3	2	1	3
18	3	4	2	3	3	3	3	4	2	2	1	1
19	1	4	3	3	1	1	1	2	1	1	2	2
20	5	5	4	4	4	4	4	4	5	5	4	3

**Figure 3:**
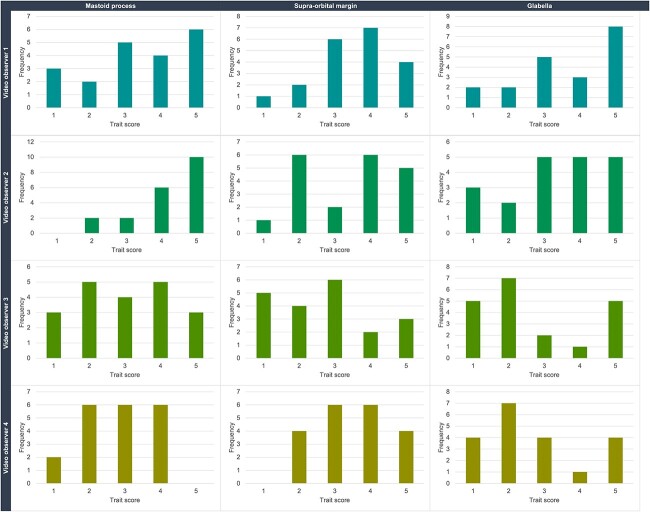
Bar charts illustrating the frequency of the cranial trait scores (1–5) per trait and per video observer.

Frequency plots of the cranial traits scores from the video observers (Figure 3) illustrate the distribution of the score data. The video observers appear to be assigning high scores more often than the lower scores.

### Video observer sex estimations

The video observer cranial trait scores (Table 3) were used to obtain sex estimations using the Walker method [17] with equation 2; the results are presented for video Observers V1–V4 in Table 4.

**Table 4:** Sex estimation results for video Observers V1–V4 calculated from Walker equation 2 [17] (prob m/f = probability male/female) and recorded sex for each cranium.

Crania code	V1			V2			V3			V4			Recorded
	Sex	Prob m (%)	Prob f (%)	Sex	Prob m (%)	Prob f (%)	Sex	Prob m (%)	Prob f (%)	Sex	Prob m (%)	Prob f (%)	
1	Male^a^	55	45	Male^a^	96	4	Male^a^	55	45	Female	20	80	Female
2	Male^a^	99	1	Male^a^	100	0	Male^a^	52	48	Female	20	80	Female
3	Male^a^	99	1	Male^a^	99	1	Female	5	95	Male^a^	52	48	Female
4	Male^a^	95	5	Male^a^	81	19	Female	5	95	Female	5	95	Female
5	Male	100	0	Male	100	0	Male	100	0	Male	100	0	Male
6	Male	100	0	Male	100	0	Male	96	4	Male	96	4	Male
7	Female	22	78	Male^a^	55	45	Female	6	94	Female	22	78	Female
8	Female	18	82	Female	5	95	Female	1	99	Female	1	99	Female
9	Male	99	1	Male	100	0	Male	100	0	Male	100	0	Male
10	Male^a^	96	4	Male^a^	96	4	Male^a^	52	48	Male^a^	52	48	Female
11	Male	100	0	Male	100	0	Male	95	5	Male	82	18	Male
12	Male	100	0	Male	100	0	Male	100	0	Male	100	0	Male
13	Male	100	0	Male	100	0	Male	100	0	Male	100	0	Male
14	Male	96	4	Male	99	1	Female	20	80	Female^a^	18	82	Male
15	Female	22	78	Male^a^	84	16	Female^a^	6	9	Female	20	80	Female
16	Male	100	0	Male	100	0	Male	100	0	Male	96	4	Male
17	Male^a^	55	45	Male^a^	52	48	Female^a^	18	82	Male^a^	55	45	Female
18	Male	52	48	Male	82	18	Female	5	95	Female^a^	18	82	Male
19	Female	1	99	Female	49	51	Male^a^	52	48	Male^a^	52	48	Female
20	Male	100	0	Male	100	0	Male	99	1	Male	96	4	Male
% correct	70			65			70			70			

a Incorrect sex assessment.

The cranial trait scores from the video observers correctly estimated the sex of the individual in 65%–70% of overall cases. Sex estimations were correctly classified in 80%–100% of cases for males (average 90%), and in 20%–45% of cases for females (average 40%).

## Discussion

This study assessed the potential for using morphoscopic methods on STL 3D cranial models in forensic anthropology. Twenty different cranial models were examined by four observers who each performed cranial trait scoring following the morphoscopic method from Walker [17]. Four video observers also performed cranial trait scoring, but on videos of the 20 models produced by Observer 1.

A high level of agreement between morphoscopic feature scores was identified, indicating good agreement between the original observers, and between the video observers scores (KCC 0.68–0.84). Despite the high agreement, higher rates of incorrect feature scoring were observed at the start of modelling (e.g. Crania 1–4), which could potentially be explained by the observers familiarizing themselves with this particular population, and its physical morphological traits. Moreover, some female crania do present with more robust traits (and *vice versa*); without knowing the variation present in the sample population, more “robust” female crania could be misinterpreted as possible male ones which is reflective of natural population variation [44]. Additionally, given the ordinal scoring system, trait scoring results alone cannot be interpreted for “accuracy”.

In several instances, observers did not use a particular score at all with certain features, such as Observer 4 with the glabella (Figure 2), or video Observer 4 with the supra-orbital margin (Figure 3). Additionally, both sets of observers frequently utilized the middle score of 3 (less so for Observer 2). These observations from the trait scoring could indicate uncertainty or a lack of confidence in utilizing the method, or stem from a wider issue around lack of applicability of the method with the population used, and/or systematic bias towards certain scores. The possible influence of age on the cranial traits was not investigated in this study, but age has previously been discounted from playing a prominent role in cranial trait expression [44].

Published studies have examined the accuracy of traditional anthropological methods of establishing sex and found varying accuracy rates to be due to either populational differences, or simply to the experience of the observers [14] and their interpretations. The scores from the glabella exhibited higher agreement between observers, in concordance with previous studies that found particular features vary in their reliability [37, 45]. The level of agreement in this study was in line with published research reported by Langley et al. [45] using crania, by Villa et al. [57] for inter-observer agreement using pelvic features, and by Lesciotto and Doershuk [46] who found moderate to substantial inter-observer agreement (using pelvic features).

Additionally, the scores from the original observers (who were scoring their own models) resulted in accurate sex estimations for 65%–95% of models using equation 2 from Walker [17]. Similarly, the video observers (who were scoring the models from Observer 1), resulted in accurate sex estimations in 65%–70% of cases. These results are lower than can be seen in other studies, such as 91.8%–92.9% [37], 93.5% using dry skulls [45] and 82.9%–85.4% reported in Walker [17]. However, the equation used in this study only included two of the five possible scoring traits. Moreover, there appears to be some bias towards male scoring for both sets of observers, as male cranial scores resulted in correct sex classifications in 80%–100% of cases. A study by Oikonomopoulou et al. [29] reported similar accuracy differences between each sex, with males providing higher classification rates (above 90%) in contrast to the female sample (22.62%–61.36%). This could be explained by the observers having more familiarity with male skeletons, an issue stemming from assessing the robusticity and gracility of the 3D models, or potentially a wider methodological issue. It is salutary that there is evidence of male bias in forensic anthropology skeletal collections [40], in traditional method development [47], and even in modern machine learning approaches [48]. New population datasets and progressive approaches are needed to overcome such biases in forensic anthropology methodologies. Observer experience has previously been shown to influence the final sex classifications [14], however observer experience with sex estimation methods was not evaluated in this study as the aim was not to evaluate accuracy but feasibility. Higher rates of correct sex estimations were obtained from the original observer trait scores than the video observer scores, and this may be explained by the familiarity of the observers with virtual anthropology, indeed Observers 1 and 2 were familiar with 3D modelling or scoring 3D crania. Training in virtual anthropology and the development of new methods that are applicable to virtual anthropology approaches are vital.

The methods used were those typically taught in forensic anthropology programmes so that each observer was familiar with the procedures of the technique. However, the observers were not familiar with applying the methods to virtual 3D models or videos, which could have affected their ability to assess the cranial features. Three of the video observers remarked that the scoring process was difficult to implement visually without the use of touch, particularly for the supra-orbital margin, which may explain some of the variation seen in the scoring. Certainly, this reflects a limitation of virtual analysis, but also poses a wider question as to the transparency of decision making in evaluative interpretation [49–52] and specifically whether more tacit information elicited from “touch” can be incorporated into a framework for transparent evaluative decision making in a forensic science context [14, 53].

Overall, the models were successfully scored for cranial traits by all observers and the models, open-source software, and video productions provided straightforward, accessible platforms for conducting remote forensic anthropology analysis. The models used in this study were STL mesh models and not volume renderings, which is an important distinction that needs to be highlighted in research applications (see section “Literature review”). To comply with local ethical requirements, it was not possible to share the STL cranial files with participants. However, it was observed that the video test with a private link worked well as a user-friendly way to temporarily remotely share the models.

Scepticism about the utility of 3D modelling has focused on the misinterpretation of modelling artefacts as pathology or trauma [26]. Indeed, the models used in this study exhibited a degree of bone loss, for example, this can be seen around Pterion with Cranium 10 (Figure 1). Although there may be instances where the 3D model does not accurately represent minor morphological features, which could potentially result in erroneous trauma and pathological identifications [26, 54], this highlights the importance of training in 3D CT modelling for forensic anthropologists. Indeed, users should understand that any missing data may be the result of CT slicing or thresholding errors, and thereby avoid misinterpreting artefacts as pathology or trauma. Moreover, these findings emphasize the need for training and establishing quality control protocols in model development, and inter-observer testing for forensic reconstructions.

The opportunity to apply the capabilities of modern imaging technologies creates new avenues of research where visual procedures in the interpretation of skeletal remains could be further enhanced using methods that may offer a less time-consuming approach (for example over manual maceration techniques), and imaging approaches facilitate remote and immediate access to scan data or virtual models. Further, using virtual anthropology and modern scan data from a living population, supports a more ethical approach than traditional osteological approaches that can avoid maceration of human remains, overhandling of skeletal collections, and colonialism and historical discriminatory practices [4]. Whilst there are associated benefits to using virtual anthropology, it is also vital to understand the underlying factors that play a role in the interpretation of current and new methods in virtual environments, including testing for the reliability and accuracy of the applicability of 3D STL cranial models in a forensic context. However, alternative ethical issues have arisen and are starting to be explored concerning the production of 3D models [55, 56]. Given the existing restrictions that can make physical access to skeletal collections difficult, there is clearly huge potential for 3D models to increase accessibility to collections through digital databases and radiographic imaging. For example, CT scanning is routinely carried out prior to autopsy in several institutes, which increases the datasets of modern populations available that may be suitable for research purposes [57] in addition to clinical datasets of living patients. Virtual anthropology offers an alternative pathway for data collection within forensic anthropology when access to traditional skeletal collections is either limited, or not possible. Therefore, traditional methods for establishing a biological profile must be further tested on virtual models to determine feasibility, as well using contemporary population datasets with contemporary discriminate function equations to improve sex estimation classifications systems. This initial study has only begun to test the feasibility of STL 3D models and highlights the need for further research to be conducted in order to establish the scope of using traditional morphoscopic methods on different skeletal elements.

The main aim of this study was to determine if it was possible to visually assess STL 3D cranial models from a modern UK population, but not to assess the accuracy of the sex estimation results. Therefore, the sex estimation results found in this study were reasonable as an indicator of sex estimation accuracy for the purpose of assessing the usability of 3D crania. Good compatibility with the sex estimation scoring method adds further weight to the robustness of the cranial models produced previously [16]. The results from this study thus add weight to the suitability of the STL 3D cranial models produced by Robles et al. [16] for morphoscopic analysis.

## Conclusions

This study has demonstrated that it was possible to apply a traditional morphoscopic forensic anthropology sex estimation method on the STL 3D cranial models produced by Robles et al. [16]. This study is the first (to our knowledge) to test the Walker method [17] on STL 3D models produced from CT data from a living UK population. Levels of inter-observer agreement were found with cranial trait scoring, and correct sex estimations ranged from 65% to 95% for both sets of observers, albeit with probable bias towards male scoring. High percentages of correct male classification were observed, with lower female classification rates.

Complementary studies are needed to assess traditional macromorphoscopic methods on other skeletal STL models such as the pubic symphysis and auricular surface from a variety of modern populations. Potential male bias in anthropology teaching and/or skeletal collections could be overcome with the utilization of modern 3D models. A comparison between interpretations made using volume renderings and those made using STL surface reconstructions would also be useful to assess whether there is any potential impact from these two digital approaches.

The ability to use free software such as 3D Slicer to view STL 3D models for morphoscopic trait scoring is important for forensic science applications in a field where funding is often very limited. It is also salient to consider how these tools will enable the development of digital databases that not only offer access to broader and more diverse populations for practitioners and researchers, but also opens up new areas of research that can be carried out with modern CT data where modern day populations are particularly relevant, as in forensic anthropology reconstructions.
